# Serum lipoprotein (a) associates with the risk of renal function damage in the CHCN-BTH Study: Cross-sectional and Mendelian randomization analyses

**DOI:** 10.3389/fendo.2022.1023919

**Published:** 2022-11-23

**Authors:** Yunyi Xie, Han Qi, Bingxiao Li, Fuyuan Wen, Fengxu Zhang, Chunyue Guo, Ling Zhang

**Affiliations:** Department of Epidemiology and Health Statistics, School of Public Health, Capital Medical University and Beijing Municipal Key Laboratory of Clinical Epidemiology, Beijing, China

**Keywords:** lipoprotein (a), lipids, renal function, Mendelian randomization, CHCN-BTH

## Abstract

**Background:**

Evidence regarding the effects of lipoprotein (a) [lp(a)] and renal function remains unclear. The present study aimed to explore the causal association of serum lp(a) with renal function damage in Chinese general adults.

**Methods:**

A total of 25343 individuals with available lp(a) data were selected from the baseline survey of the Cohort Study on Chronic Disease of Communities Natural Population in Beijing, Tianjin, and Hebei (CHCN-BTH). Five renal function indexes [estimated glomerular filtration rate (eGFR), serum creatinine (Scr), blood urea nitrogen (BUN), uric acid (UA), high-sensitivity C-reactive protein(CRPHS)] were analyzed. The restricted cubic spline (RCS) method, logistic regression, and linear regression were used to test the dose-response association between lp(a) and renal function. Stratified analyses related to demographic characteristics and disease status were performed. Two-sample Mendelian randomization (MR) analysis was used to obtain the causal association of lp(a) and renal function indexes. Genotyping was accomplished by MassARRAY System.

**Results:**

Lp(a) levels were independently associated with four renal function indexes (eGFR, Scr, BUN, CRPHS). Individuals with a higher lp(a) level had a lower eGFR level, and the association with Scr estimated GFR was stronger in individuals with a lower lp(a) level (under 14 mg/dL). . The association was similar in individuals regardless of diabetes or hypertension. MR analysis confirmed the causal association of two renal function indexes (Scr and BUN). For MR analysis, each one unit higher lp(a) was associated with 7.4% higher Scr (P=0.031) in the inverse-variance weighted method. But a causal effect of genetically increased lp(a) level with increased eGFR level which contrasted with our observational results was observed.

**Conclusion:**

The observational and causal effect of lp(a) on Scr and BUN were founded, suggesting the role of lp(a) on the risk of renal function damage in general Chinese adults.

## Introduction

Chronic kidney disease (CKD) affects 6–19% of the population in China (1, 2). One cross-sectional survey reported that the prevalence of glomerular filtration rate (GFR) less than 60 ml/min/1.73 m2 was about 2% (1). CKD had a major effect on the cause of morbidity and mortality and is a critical complication for cardiometabolic diseases (1). The mortality and cardiovascular outcomes are strongly influenced by kidney disease in the general population, especially in high-risk diabetic or hypertensive populations (2). Decreased GFR is an important renal marker for the diagnosis of CKD (3).

Lipoprotein(a) [lp(a)] is a plasma lipoprotein contain a cholesterol-rich LDL particle with one molecule of apolipoprotein B100 and an extra apolipoprotein(a) (4). Extremely broad and skewed distribution can be seen on lp(a) which is largely influenced by genetic variants at the LPA locus (5). Lp(a) is a well-known cardiovascular risk factor. Besides, studies indicate that the kidney is involved in the catabolism of lp(a) and that elevated lp(a) levels are associated with kidney disease (6). Several studies have suggested that renal function is one of the nongenetic factors influencing plasma lp(a) levels (6). However, the effect of lp (a) on renal function damage has not been fully disclosed. One Meta-Analysis suggested that higher serum lp(a) was associated with the risk of diabetic nephropathy (7). A prospective study including 6257 adults indicated that high lp(a) levels interacted with diabetes were risk factors for reduced renal function (8) while the absence of genetic data for lp(a) and was limited to middle-aged and older adults. A larger sample size study for the validation of the previous results and assessment of the causal association in young to older adults was needed.

In the present study, data for 25343 individuals within the baseline survey of the Cohort Study on Chronic Disease of Communities Natural Population in Beijing, Tianjin, and Hebei (CHCN-BTH) were used to analyze the association of lp(a) level to renal function damage. Stratified analyses for demographic characteristics and disease status were performed to explore the effect modification on the associations between lp(a) and renal function damage. Furthermore, the two-sample mendelian randomization (MR) approach was used to test the causal association between genes-determined lp(a) level and indexes of renal function.

## Methods

### Study subjects and sample collection

A total of 25343 individuals with available lp(a) data were selected from the baseline survey of the Cohort Study on Chronic Disease of Communities Natural Population in Beijing, Tianjin, and Hebei (CHCN-BTH, Registration number: ChiCTR1900024725) study was selected in this study, detailed information of which has been published previously (9–11). Briefly, the CHCN-BTH study was a community population-based chronic disease study aimed to analyze the risk factors for major chronic diseases. The CHCN-BTH study was approved by the Ethics Committee of the Centre of Disease Control (IRB2017-003, CYCDPCIRB-20170830–1) and Capital Medical University (2018SY81), and written informed consent was gained from each individual before the study. Among 33391 recruiters, objects were excluded according to the following criteria (1): non-Han ethnicity (n=3366) (2); had missing data in the lp(a) (3); had missing data in the key demographic variables (n=4682). A total of 25343 recruiters were finally included in the current study.

### Biochemical measurements

All blood biochemical measurements were conducted utilizing the Beckman Coulter chemistry analyzer AU5800 in the clinical laboratory of Beijing Hepingli Hospital, and all procedure was blinded to the study researchers. The lp(a) levels in the serum samples were measured using a particle-enhanced turbidimetric immunoassay, lp(a) Latex [DAIICHI] (Sekisui Diagnostic Ltd, Japan). The assay principle is that lp(a) reacts with anti-human lp(a) mouse monoclonal antibody-sensitized latex beads to motivate agglutination of the beads. The lp(a) level is estimated by calculating the variant of turbidity because of the agglutination reaction. Lp(a) latex standard sera L, M, and H are utilized for calibration, and the standard material is purified lp(a) (in-house reference standard). The automatic biochemical meter of Beckman from Japan (AU5800) was utilized for the biochemical test. fasting blood plasma glucose (FPG) was measured by the hexokinase method, and the low-density lipoprotein cholesterol (LDL-C) was determined by the selective solubilization method (low-density lipid cholesterol test kit). The concentrations of total cholesterol (TC), triglyceride (TG), and high-density lipoprotein cholesterol (HDL-C) were measured by enzymatic assays. The fasting serum creatinine (Scr) level was measured by using the picric acid method on an autoanalyzer. Uric acid was measured by the uricase colorimetric method, and urea was measured by the urease UV rate method. The level of high-sensitivity C-reactive protein (CRPHS) was measured *via* the immunoturbidimetric.

### Assessment of reduced renal function

GFR (expressed in milliliters per minute per 1.73 square meters, ml/min/1.73 m^2^) was evaluated by the 2009 Chronic Kidney Disease Epidemiology Collaboration (CKD-EPI) model which is the most accurate method for estimating GFR for varied populations (12). The formulas were: eGFR=a×(Scr/b)c×(0.993)age; The value of a adopts the following values according to gender and race (1): Black: Female = 166, Male = 163 (2), Other races: female = 144, male = 141; the b value is different according to the gender as follows (1): Female = 0.7 (2),Male = 0.9; The value of c is as follows according to age and Scr value (1): Female: Scr ≤ 0.7 mg/dL = -0.329, Scr >0.7 mg/dL = -1.209 (2), Men: Scr ≤ 0.7 mg/dL= -0.411, Serum creatinine>0.7 mg/dL = -1.209. Normal eGFR value range from 120 to 138 ml/min/1.73 m^2^. eGFR under 60 ml/min/1.73 m^2^ was defined as reduced renal function, eGFR between 60-89 ml/min/1.73 m^2^ was defined as mildly decreased renal function, and GFR under 90 ml/min/1.73 m^2^ were defined as slightly renal function damage (13–15).

### Covariates and definitions of diabetes and hypertension

Potential confounders were included based on the previous study on CKD (16). The covariates included age (years, as a continuous covariate), gender (female/male), residential place (urban/rural), education degree (primary school/secondary/senior school/undergraduate), smoking status (current smoking/not current smoking), alcohol taking status (current drinking/not current drinking), exercise (5-7 days a week/1-4 days a week/less than 1 day a week), body mass index (BMI, kg/m2, as a continuous variable), high-density lipoprotein cholesterol (HDL-C, mmol/L, as a continuous variable), diabetes mellitus (yes/no), hypertension (yes/no). Individuals were diagnosed having hypertension if a measured SBP≥140 mmHg or DBP≥90 mmHg, and/or had self-reported physician-diagnosed hypertension or antihypertensive drug-taking history according to the Chinese Guidelines for the Management of Hypertension (17). Diabetes was defined as having a fasting plasma glucose (FPG)≥7.0 mmol/L, and/or having a self-reported diagnosis of diabetes (18).

### Statistical methods

Individuals were divided into five categories based on the quartiles of serum lp(a) level (50th, 75th, 90th, 95th, 100th). These predefined categories were utilized throughout the study. Continuous variables are presented as mean ± SD for the normality distributions variable and median ± interquartile range (IQR) for the skewed distributions variable. Categorical variables were presented as the count and percentage. One-way ANOVA was conducted for continuous variables and Chi-square (χ2) analysis was conducted for categorical variables for multigroup comparisons. Variables with non-normal distribution were logarithmically transformed. P for trend was determined by utilizing the Cochran-Armitage trend test for the association between continuous and categorical variables with the five lp(a) categories. Spearman correlations were used to analyze the correlation between renal function indexes and their risk factors. The restricted cubic spline (RCS) method was utilized to test the dose-response association between lp(a) and renal function with 5 knots. Logistic regression models were performed to estimate the associations between lp(a) and reduced renal function (eGFR under 90 and eGFR under 60). Linear regression models were performed to estimate the associations between lp(a) and indexes of renal function (eGFR, Scr, BUN, UA, and CRPHS). Furthermore, we conducted the stratified analysis on the association between lp(a) levels and renal function according to age (as category variable), gender, baseline diabetes, and hypertension. Multivariable adjustments included age, gender, residential place, education, exercise frequency, smoking status, alcohol taking, BMI, HDL-C, hypertension, diabetes, Scr, BUN, UA, and CRPHS. The statistical analysis was operated in R software (version 3.4.4) and a two-sided P <0.05 was considered statistically significant.

### Genotyping

Genomic DNA was isolated from 200 μL of a suspension of EDTA-anticoagulated peripheral blood leukocytes utilizing the Magnetic Beads Whole Blood Genomic DNA Extraction Kit by using an automatic nucleic acid extraction apparatus (BioTeke, Beijing, China). A NanoDrop 2000 spectrophotometer (Thermo Fisher Scientific, Waltham, MA, USA) was conducted to measure the concentration and purity of the extracted DNA. All SNPs were genotyped utilizeing the high-throughput sequencing method on the Sequenom Mass ARRAY Platform (Sequenom, San Diego, CA, USA).

### Mendelian randomization

Lp(a)-related tags SNP were analyzed in our previous study (9). Thirteen SNPs with GWAS evidence were selected for our study. Five SNPs (rs1018234, rs2048327, rs41269133, rs520829, and rs641990) in SLC22A3 gene, three SNPs (rs6415084, rs7765781, and rs7770628) in LPA gene, two SNPs (rs429358 and rs7412) in APOE gene, one SNP rs5930 in LDLR gene.

Summary statistics for the associations of each SNP with indexes of renal function in the East Asian population were assessed from the openly published GWAS data. Data sources for the outcome prototypes included in the present MR study were detailed in **Supplementary Table S4**. The GWAS summary statistics for eGFR (19), Scr (20), Cystatin C (19), BUN (20), UA (20), and CRPHS (20) were generated utilizing UK Biobank and Pan-UKB which were available from the MRC-IEU OpenGWAS database (https://gwas.mrcieu.ac.uk/). The script of the MR analysis performed in our research is accessible in the GitHub repository of the ‘TwoSampleMR’ R package (https://github.com/MRCIEU/TwoSampleMR/).

The MR estimates for each outcome were analyzed utilizing the inverse variance weighted (MR-IVW) method. The random-effects meta-analysis method combines the Wald ratio estimates of the causal effect accessed from each including SNPs was used for the MR-IVW method (21). Sensitivity analyses were conducted with MR–Egger, MR weighted median, and MR weighted mode. and heterogeneity test (22). In addition, the MR-Egger regression method was utilized to explore the pleiotropy, and heterogeneities between the SNPs were evaluated using Cochran’s Q-statistics, and the leave-one-out analysis was conducted to test the effect of outlying or pleiotropic SNP (22). The mRnd power calculator (https://cnsgenomics.shinyapps.io/mRnd/) (23) was performed to calculate the statistical power for MR. We estimated the effect of Scr and lp(a) level, and 80% statistical power was achieved, given the sample size and lp(a) variance explained by the genetic instruments.

## Results

### Baseline characteristics of the study population

A total of 25,343 individuals were recruited and finished the baseline survey. The mean age of the study population was 50.7 years (SD 14.4); 13,774 (54.4%) were female; the mean lp(a) level was 18.9 (SD 20.5) mg/dl; the mean eGFR was 105.1 (SD 32.2) ml/min/1.73 m^2^; the prevalence of eGFR with slightly declining (under 90) was 15.8%, with mildly declining (eGFR under 60) was 0.6%; the mean BUN level was 5.1 (SD 1.7) mmol/l; mean UA level was 321.9 (SD 91.8) μmol/l. Baseline characteristics of study recruiters according to the category of baseline serum lp (a) level were shown in **Table 1**. The recruiters with the highest quartile of lp (a) had a higher mean age, female proportion, Scr levels, BUN levels, frequency of living in rural areas, undergraduate rate, exercise frequency, FPG levels, TC levels, HDL-C levels, LDL-C levels, diabetes mellitus prevalence, CHD, and stroke prevalence, compared with the lowest quartile of lp (a) (all P for trend <0.05). The recruiters with the highest quartile of lp (a) had a lower eGFR, current smoking rate, alcohol-taking rate, BMI, TG levels, and hypertension prevalence, compared with the lowest quartile of lp (a) (all P for trend <0.05).

**Table 1:** Baseline characteristics for all individuals and by Lp(a) percentile category.

Variables	All individuals	Lp (a) Percentiles						P for trend
		[0, 25]	[25, 50)	[50, 75)	[75, 90)	[90, 95)	[95, 100]	
N	25343 (100)	6327 (25.0)	6336 (25.0)	6343 (25.0)	3800 (15.0)	1267 (5.0)	1268 (5.0)	
Lp (a), mg/dl	18.85 ± 20.47	3.53 ± 1.40	8.68 ± 1.73	16.90 ± 3.36	31.76 ± 5.57	50.77 ± 4.83	85.32 ± 23.24	<0.001
Scr, μmol/l	64.27 ± 16.37	62.83 ± 13.96	64.05 ± 14.21	65.09 ± 14.67	65.05 ± 14.45	65.76 ± 14.79	64.59 ± 14.42	<0.001
eGFR, ml/min/1.73 m^2^	105.09 ± 32.17	107.70 ± 16.24	105.15 ± 16.11	104.09 ± 16.39	103.78 ± 16.49	103.47 ± 16.13	102.41 ± 16.57	<0.001
eGFR under 90, ml/min/1.73 m^2^	4012 (15.8)	776 (12.3)	970 (15.3)	1093 (17.3)	688 (18.1)	226 (17.8)	259 (20.4)	<0.001
eGFR under 60, ml/min/1.73 m^2^	152(0.6)	23 (0.4)	29 (0.5)	51 (0.8)	24 (0.6)	11 (0.9)	14 (1.1)	0.001
BUN, mmol/l	5.05 ± 1.67	5.00 ± 1.61	5.07 ± 2.07	5.07 ± 1.41	5.05 ± 1.39	5.05 ± 1.47	5.21 ± 1.81	0.004
UA, umol/l	321.90 ± 90.75	321.38 ± 90.98	321.62 ± 91.69	323.19 ± 91.01	322.55 ± 89.20	324.07 ± 89.14	315.48 ± 89.71	0.9016
CRPHS, mg/l	1.91 ± 2.69	1.79 ± 2.40	1.90 ± 2.72	1.99 ± 2.86	1.91 ± 2.73	1.95 ± 2.80	2.00 ± 2.80	0.154
Age, years	50.69 ± 14.44	50.11 ± 13.84	50.75 (14.39)	50.72 ± 14.61	51.11 ± 14.86	50.56 ± 14.77	51.90 ± 15.05	<0.001
Female sex	13774 (54.4)	3255 (51.4)	3472 (54.8)	3468 (54.7)	2099 (55.2)	696 (54.9)	784 (61.9)	<0.001
Area, urban	19962 (78.8)	4639 (73.4)	5003 (79.0)	5107 (80.5)	3100 (81.5)	1060 (83.6)	1053 (83.1)	<0.001
Education								
primary	2838 (11.2)	767 (12.1)	759 (12.0)	695 (11.0)	384 (10.1)	105 (8.3)	128 (10.1)	<0.001
secondary	7953 (31.4)	2267 (35.8)	2043 (32.2)	1822 (28.7)	1108 (29.1)	354 (27.9)	359 (28.3)	
senior	6042 (23.8)	1612 (25.5)	1504 (23.7)	1502 (23.7)	877 (23.1)	282 (22.3)	265 (20.9)	
undergraduate	8510 (33.6)	1681 (26.6)	2030 (32.0)	2324 (36.6)	1433 (37.7)	526 (41.5)	516 (40.7)	
Physical activity								
Low	18883 (74.5)	4771 (75.4)	4643 (73.3)	4710 (74.3)	2829 (74.4)	977 (77.1)	953 (75.2)	0.136
Intermediate	5541 (21.9)	1294 (20.5)	1460 (23.0)	1421 (22.4)	836 (22.0)	255 (20.1)	275 (21.7)	
High	919 (3.6)	262 (4.1)	233 (3.7)	212 (3.3)	137 (3.6)	36 (2.8)	39 (3.1)	
Exercise								
5-7 d/w	11455 (45.2)	2750 (43.5)	2897 (45.7)	2908 (45.8)	1702 (44.8)	596 (47.0)	602 (47.5)	<0.001
1-4 d/w	8512 (33.6)	2036 (32.2)	2121 (33.5)	2133 (33.6)	1345 (35.4)	422 (33.3)	454 (35.9)	
<1 d/w	5376 (21.2)	1541 (24.4)	1318 (20.8)	1302 (20.5)	755 (19.9)	250 (19.7)	210 (16.6)	
Current smoking	6038 (23.8)	1753 (27.7)	1492 (23.6)	1484 (23.4)	822 (21.6)	281 (22.2)	206 (16.3)	<0.001
Alcohol taking	9268 (36.6)	2535 (40.1)	2253 (35.6)	2296 (36.2)	1329 (35.0)	453 (35.8)	402 (31.7)	<0.001
BMI, kg/m^2^	25.36 ± 3.64	25.69 ± 3.67	25.42 ± 3.58	25.19 ± 3.53	25.14 ± 3.52	24.92 ± 3.42	24.80 ± 3.36	<0.001
FPG, mmol/L	5.84 ± 1.61	5.86 ± 1.76	5.80 ± 1.51	5.83 ± 1.58	5.85 ± 1.60	5.86 ± 1.53	5.92 ± 1.67	<0.001
TG, mmol/L	1.60 ± 1.07	1.81 ± 1.36	1.61 ± 1.02	1.51 ± 0.92	1.47 ± 0.89	1.48 ± 0.95	1.49 ± 0.84	<0.001
TC, mmol/L	5.12 ± 1.04	4.93 ± 1.03	5.07 ± 1.01	5.17 ± 1.03	5.24 ± 1.04	5.28 ± 1.03	5.49 ± 1.06	<0.001
HDL-C, mmol/L	1.36 ± 0.36	1.31 ± 0.37	1.35 ± 0.35	1.38 ± 0.35	1.39 ± 0.35	1.41 ± 0.35	1.44 ± 0.37	<0.001
LDL-C, mmol/L	3.02 ± 0.86	2.81 ± 0.87	3.00 ± 0.82	3.09 ± 0.85	3.15 ± 0.85	3.18 ± 0.85	3.34 ± 0.88	<0.001
Hypertension	6978 (27.5)	1927 (30.5)	1715 (27.1)	1686 (26.6)	1002 (26.4)	314 (24.8)	334 (26.4)	<0.001
Diabetes mellitus	2725 (10.8)	787 (12.4)	669 (10.6)	642 (10.1)	372 (9.8)	122 (9.6)	133 (10.5)	0.003
CVD (CHD+Stroke)	1949(7.7)	483 (7.6)	457 (7.2)	494 (7.8)	285 (7.5)	108 (8.5)	122 (9.6)	<0.001

Data are n (%) for categorical variables or mean ± SD for continuous variables.

Scr, serum creatinine; eGFR, estimated glomerular filtration rate; BUN, blood urea nitrogen; UA, uric acid; CRPSH, high-sensitivity C-reactive protein; BMI, body mass index; FPG, fasting blood plasma glucose; TG, Triglycerides; TC, Total cholesterol; HDL-C, high-density lipoprotein cholesterol; LDL-C, low-density lipoprotein cholesterol; CVD, cardiovascular disease; CHD, coronary heart disease.

### Correlation between the indexes of renal function and its risk factors

**Figure 1:**
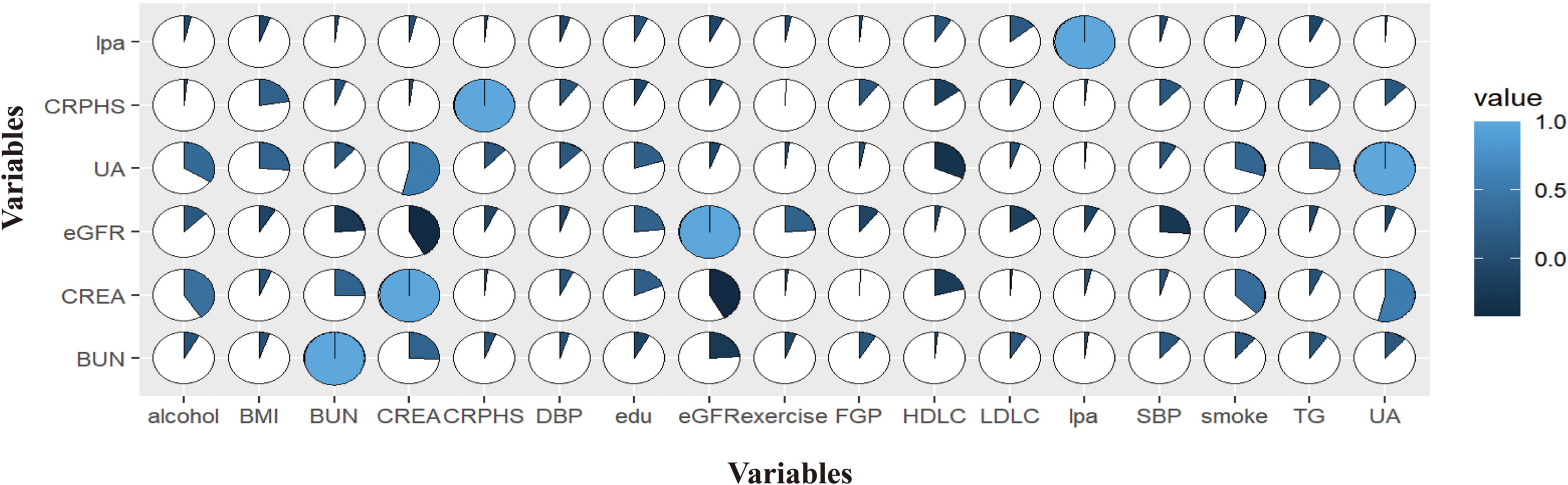
Correlations of indexes of renal function and it’s risk factors.

**Figure 2:**
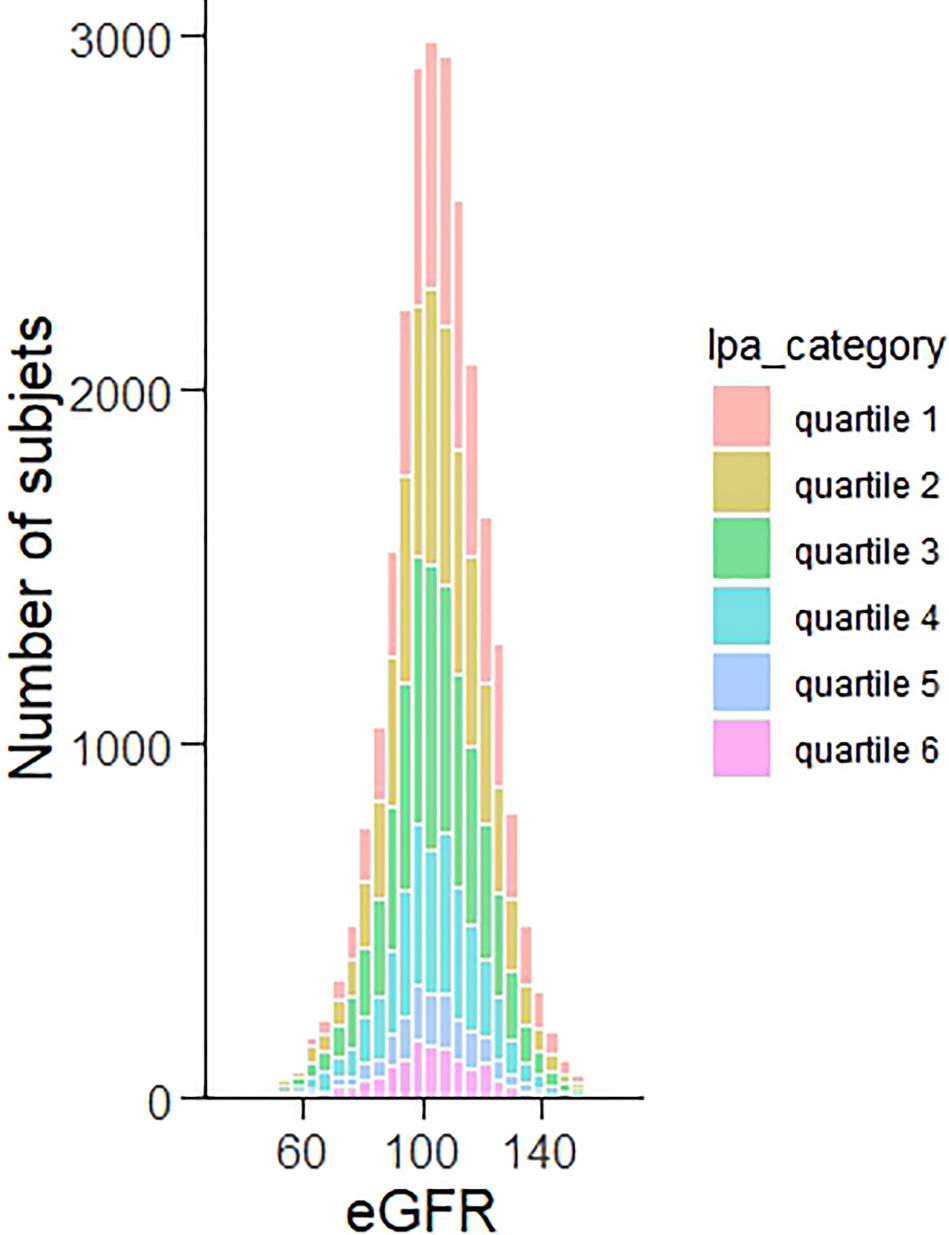
Distribution of eGFR grouped by lp(a) categories in Chinese Han population.

The correlation between the index of renal function and its risk factors was shown in **Figure 1**. Lp(a) levels, age, gender, BUN, UA, LDL-C, and education, negatively correlated with the eGFR. Living area, CRPHS, TG, HDL-C, exercising frequency, smoking status, alcohol taking, SBP, FBG, and BMI positively correlated with the eGFR. **Figure 2** depicted the broad and skewed distribution of eGFR in recruiters of the Chinese Han population grouped by lp(a) levels. The distribution of eGFR showed a normal distribution .

### Non-linear effects of lp(a) on the indexes of renal function

**Figure 3:**
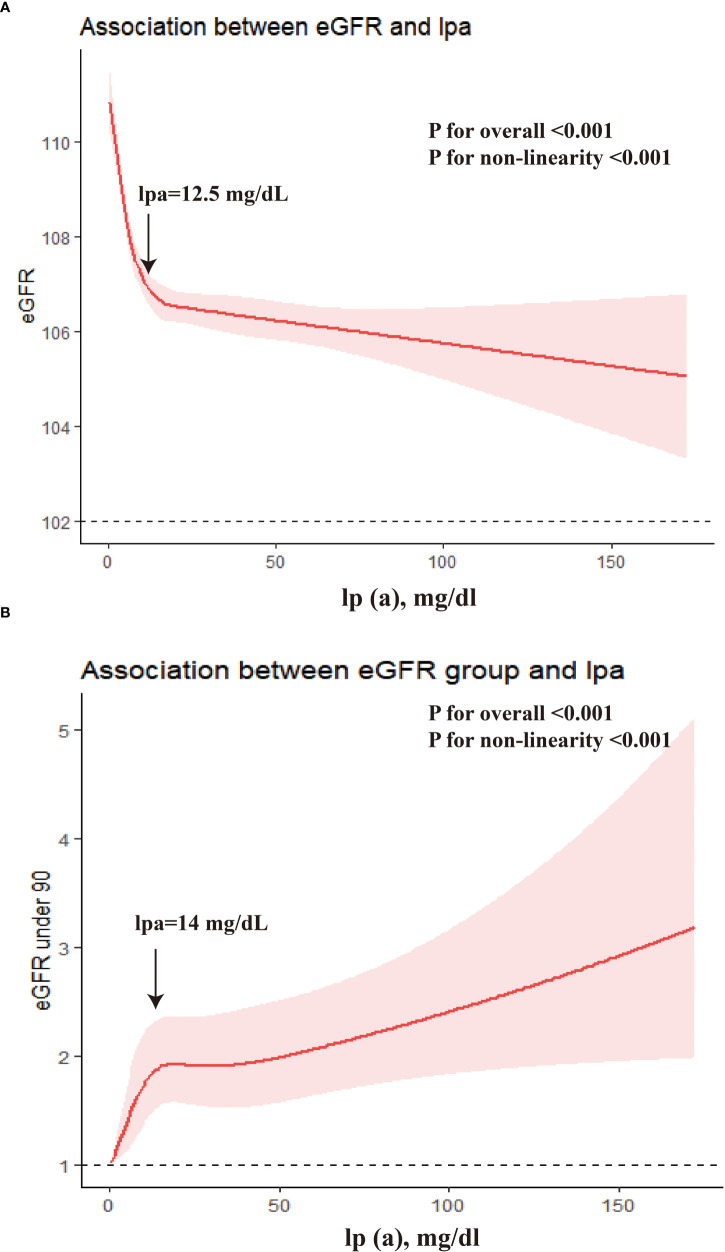
The dose-response associations between lp(a) and eGFR. **(A)** β for the association of lp(a) and eGFR as continuous variable; **(B)** Odds ratio for the association of lp(a) and eGFR under 90.

We used an RCS model with 5 knots to simulate the relationship between lp(a) and the level of eGFR and the risk of slightly reduced renal function (**Figure 3**). Multivariable-adjusted RCS analyses declared that there were significant non-linearity associations of lp(a) level with eGFR as a continuous variable, and with the prevalence of slightly reduced renal function (eGFR under 90) (all P for nonlinear<0.001). With the continuous change of lp(a) level, the association strength of eGFR and risk of slightly reduced renal function (eGFR under 90) decreased nonlinearly. The association between eGFR level and lp(a) was stronger in the individual with lp(a) level under 12.5 mg/dL, and the association between slightly reduced renal function and lp(a) level was stronger in the individual with lp(a) level under 14 mg/dL.

There was significant overall associations of lp(a) level with Scr (P<0.001), BUN (P=0.045), CRPHS (P<0.001), and significant non-linearity association with Scr (P for nonlinear<0.001), BUN (P for nonlinear=0.037), and CRPHS (P for nonlinear<0.001). No significant overall associations were observed between lp(a) level and mildly renal declining (eGFR under 60), and UA (all P for overall>0.05). Results were shown in **Supplementary Figure S1**.

### Associations of lp (a) concentrations with risk of reduced renal function

As shown in **Table 2**, multivariable logistic regression analysis showed that each 1-unit increase in log10-lp (a) (mg/dl) was associated with 1.006-folds (95%CI 1.004-1.007, P<0.001) increased risk of slightly reduced renal function; Compared to the lowest quartile of lp(a), the prevalence of slightly reduced renal in the second quartile, the third quartile, the fourth quartile, the fifth quartile, and the sixth quartile were increased by 23.5% (OR=1.235, 95%CI 1.095, 1.390), 41.6% (OR=1.416, 95%CI 1.259, 1.594), 46.3% (OR=1.463, 95%CI 1.280, 1.672), 47.2% (OR=1.472, 95%CI: 1.211, 1.788), and 60.3% (OR=1.603, 95%CI: 1.325, 1.938) after adjustment for age, gender, region, BMI, HDL-C, LDL-C, TG, TC, current smoking status, alcohol taking, exercise, education status, hypertension, diabetes mellitus, BUN, UA, CRPHS (model 2), respectively (P for trend <0.001).

**Table 2:** Association of serum lp(a) concentrations with risk of renal function damage.

		Model 1		Model 2	
	Cases, N (%)	OR (95%CI)	P	OR (95%CI)	P
**Continuous**					
lp (a)	4012 (15.8)	1.006 (1.004, 1.007)	<0.001	1.006 (1.004, 1.008)	<0.001
**Categorical**					
<25%	776 (12.3)	Ref.	–	Ref.	–
25 to <50%	970 (15.3)	1.250 (1.116, 1,399)	<0.001	1.235 (1.095, 1.390)	0.001
50 to <75%	1093 (17.2)	1.459 (1.305, 1.631)	<0.001	1.416 (1.259 1.594)	<0.001
75 to <90%	688 (18.1)	1.474 (1.299, 1.672)	<0.001	1.463 (1.280, 1.672)	<0.001
90 to <95%	226 (17.8)	1.511 (1.256, 1.819)	<0.001	1.472 (1.211, 1.788)	<0.001
≥95%	259 (20.4)	1.664 (1.390, 1.991)	<0.001	1.603 (1.325, 1.938)	<0.001
**P for trend**			<0.001		<0.001

Model 1 was adjusted for age, gender, area, education, and BMI.

Model 2 was adjusted for age, gender, region, BMI, HDL-C, LDL-C, TG, TC, current smoking status, alcohol taking, exercise, education status, hypertension, diabetes mellitus, BUN, UA, CRPHS.

Furthermore, stratified analysis was conducted by baseline hypertension or diabetes status (**Tables 3**, **4**). The results showed that the associations between serum lp (a) level and risk of slightly reduced renal function were consistent with the total recruiters in individuals with or without hypertension or individuals with or without diabetes. No significant association was observed with the prevalence of slightly reduced renal in the second quartile, and the fourth quartile, compared to the lowest quartile of lp(a)(P>0.05) in the hypertension individuals. No significant association was observed with the prevalence of slightly reduced renal in the second quartile and fifth quartile, compared to the lowest quartile of lp(a)(P>0.05) in the diabetes individuals. No interactions have been observed between high lp(a) level (>30 mg/dL) and diabetes or hypertension on the risk of reduced renal function (eGFR under 90) (P for interaction>0.05) after adjusting for other risk factors (result not shown).

**Table 3:** Association of lp(a) levels with risk of renal function damage stratified by hypertension.

	Hypertension			Normotension		
	Cases, N (%)	OR (95%CI)	P	Cases, N (%)	OR (95%CI)	P
**Continuous**						
lp (a)	4012 (15.8)	1.004 (1.001, 1.007)	0.011		1.006 (1.004, 1.009)	<0.001
**Categorical**						
<25%	420 (21.8)	Ref.	–	356 (8.1)	Ref.	–
25 to <50%	432 (25.2)	1.107 (0.931, 1.318)	0.250	538 (11.6)	1.394 (1.195, 1.625)	<0.001
50 to <75%	526 (31.2)	1.407 (1.186, 1.670)	<0.001	567 (12.2)	1.499 (1.284, 1.750)	<0.001
75 to <90%	314 (31.3)	1.307 (1.072, 1.594)	0.008	374 (13.4)	1.608 (1.354, 1.750)	<0.001
90 to <95%	93 (29.6)	1.166 (0.862, 1.578)	0.319	133 (13.9)	1.771 (1.395, 2.249)	<0.001
≥95%	117 (35.0)	1.458 (1.087, 1.955)	0.012	142 (15.2)	1.809 (1.430, 2.289)	<0.001
**P for trend**				0.001		<0.001

Adjusted for adjusted for age, gender, region, BMI, HDL-C, LDL-C, TG, TC, current smoking status, alcohol taking, exercise, education status, diabetes mellitus, BUN, UA, CRPHS.

**Table 4:** Association of lp(a) levels with risk of renal function damage stratified by diabetes.

		Diabetes			No diabetes	
	Cases, N (%)	OR (95%CI)	P	Cases, N (%)	OR (95%CI)	P
**Continuous**						
lp (a)	152 (0.6)	1.006 (1.001, 1.011)	0.010		1.006 (1.004, 1.008)	<0.001
**Categorical**						
<25%	155 (19.7)	Ref.	–	621 (11.2)	Ref.	–
25 to <50%	158 (23.6)	1.078 (0.813, 1.430)	0.602	812 (14.3)	1.286 (1.135, 1.457)	<0.001
50 to <75%	174 (27.1)	1.384 (1.044, 1.836)	0.024	919 (16.1)	1.471 (1.299, 1.666)	<0.001
75 to <90%	107 (28.8)	1.402 (1.011, 1.944)	0.043	581 (16.9)	1.494 (1.298, 1.719)	<0.001
90 to <95%	35 (28.7)	1.370 (0.834, 2.251)	0.214	191 (16.7)	1.533 (1.252, 1.876)	<0.001
≥95%	47 (35.3)	2.055 (1.298, 3.254)	0.002	212 (18.7)	1.613 (1.321, 1.969)	<0.001
**P for trend**			0.001			<0.001

Adjusted for adjusted for age, gender, region, BMI, HDL-C, LDL-C, TG, TC, current smoking status, alcohol taking, exercise, education status, hypertension, BUN, UA, CRPHS.

Corresponding analyses for the other categories of 20^nd^, 40^th^, 60^th,^ and 80^th^ revealed similar results for all investigated endpoints (**Supplementary Table S1**). Compared to the lowest quartile of lp(a), the prevalence of slightly reduced renal in the second quartile, the third quartile, the fourth quartile, and the fifth quartile was increased by 27.1% (OR=1.271, 95%CI 1.117, 1.447), 38.8% (OR=1.388, 95%CI 1.220, 1.580), 64.1% (OR=1.641, 95%CI 1.445, 1.865) and 60.3% (OR=1.603, 95%CI: 1.409, 1.823) after adjustments, respectively (P for trend <0.001).

### Associations of serum lp (a) level with the indexes of renal function

**Figure 4:**
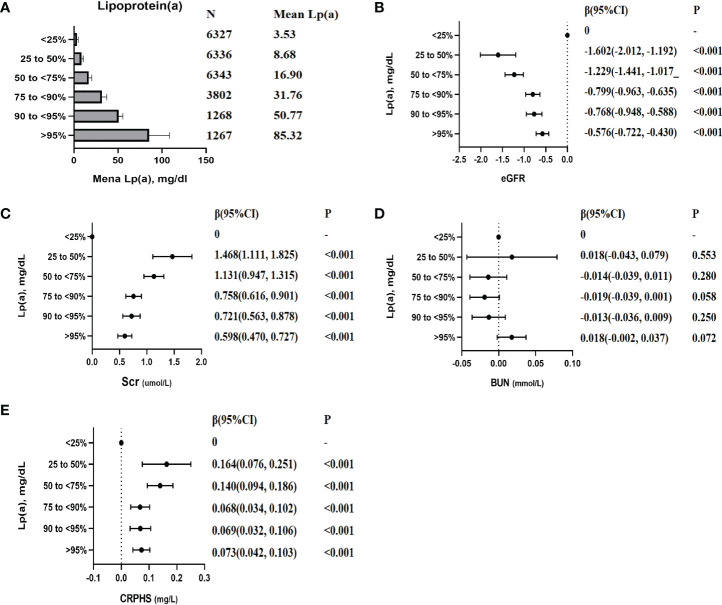
The association between lp(a) (categorical variables) with the indexes of renal function. Each model was adjusted by potential confounders except for itself. β (95% CI), beta with 95% confidence interval. **(A)**: Mean lp(a) levels of each categories; **(B)**: β (95% CI) for the association between eGFR and lp(a) levels; **(C)**: β (95% CI) for the association between Scr and lp(a) levels; **(D)**: β (95% CI) for the association between BUN and lp(a) levels; **(E)**: β (95% CI) for the association between CRPHS and lp(a) levels;.

Compared with the lowest quartile of lp(a), the eGFR decreased by 1.602 ml/min/1.73 m^2^ (P < 0.001), 1.229 ml/min/1.73 m^2^ (P < 0.001), 0.799 ml/min/1.73 m^2^ (P < 0.001), 0.768 ml/min/1.73 m^2^ (P < 0.001), and 0.576 ml/min/1.73 m^2^ (P < 0.001); the Scr increased by 1.468 μmol/l (P < 0.001), 1.131 μmol/l (P < 0.001), 0.758 μmol/l (P < 0.001), 0.721 μmol/l (P < 0.001), and 0.598 μmol/l (P < 0.001); increased CRPHS by 0.164 mg/l (P < 0.001), 0.140 mg/l (P < 0.001), 0.068 mg/l (P < 0.001), 0.069 mg/l (P < 0.001), and 0.073 mg/l (P < 0.001), with individuals in the second quartile, the third quartile, the fourth quartile, the fifth quartile, and the sixth quartile, respectively (**Figure 4**). No significant association was observed between lp(a) and BUN (P > 0.05). Sensitive analyses were conducted by excluding individuals with eGFR under 90 or with eGFR under 60. After excluding these individuals, the association between lp(a) level and indexes of renal function was consistent with the above results, respectively (**Supplementary Table S2-3**).

After stratified analysis, the associations of serum lp (a) level with the indexes of renal function had no gender and age difference (**Supplementary Table S4-5**), except for BUN. No significant association was found between lp(a) categories and BUN level except in females, or older adults aged older than 55 years. After stratified by diabetes or hypertension status, the effects of serum lp (a) levels on the indexes of renal function were consistent among individuals with diabetes or without diabetes and individuals with hypertension or hypertension, except for BUN (**Supplementary Figure S2-5**).

Corresponding analyses for the other categories of 20^nd^, 40^th^, 60^th,^ and 80^th^ revealed similar results for all investigated endpoints in total participants (**Supplementary Figure S6**).

### Association of genetic variants with lp(a)

Twenty-eight SNPs were compatible with Hardy-Weinberg equilibrium (HWE) (P > 0.05), and the result was shown in our previous published paper (Xia, 2021). Sixteen SNPs were excluded due to a lack of GWAS-level evidence, or effect sizes of opposite signs. Finally, this study included 13 SNPs as instrumental variables (4 SNPs in the SLC22A3 gene, 3 SNPs in the LPA gene, 2 SNP in the APOE gene, and 3 SNPs in undefined pathways (**Table 5**).

**Table 5:** GWAS identified SNPs Associated with lp (a) Level.

SNP	chr	pos	gene	EA	OA	MAF	log_OR	SE	P	N_Samples
rs1018234	6	160000000	SLC22A3	T	C	0.38	-0.046	0.019	1.600E-02	1256
rs1406888	6	161000000	–	T	C	0.54	0.050	0.018	5.627E-03	1256
rs2048327	6	160000000	SLC22A3	C	T	0.45	0.046	0.018	1.300E-02	1256
rs41269133	6	161000000	SLC22A3	C	T	0.23	-0.235	0.021	2.920E-28	1256
rs429358	19	44908684	APOE	C	T	0.08	-0.084	0.033	1.200E-02	1256
rs520829	6	160000000	SLC22A3	G	T	0.32	-0.051	0.020	9.000E-03	1256
rs56393506	6	161000000	–	T	C	0.18	0.309	0.022	9.080E-41	1256
rs5930	19	11113589	LDLR	A	G	0.37	-0.036	0.019	6.100E-02	1256
rs6415084	6	161000000	LPA	T	C	0.17	0.272	0.023	3.510E-30	1256
rs641990	6	160000000	SLC22A3	A	G	0.39	-0.033	0.019	8.600E-02	1256
rs7412	19	44908822	APOE	T	C	0.08	-0.062	0.035	7.400E-02	1256
rs7765781	6	161000000	LPA	C	G	0.40	-0.180	0.017	5.450E-24	1256
rs7770628	6	161000000	LPA	C	T	0.18	0.304	0.022	5.090E-40	1256

SNP, single nucleotide polymorphism; EA, effect allele; OA, other allele; MAF, minor allele frequency; SE, standard error.

Adjusted for age and gender.

### Causal association of lp(a) with the indexes of renal function

**Figure 5:**
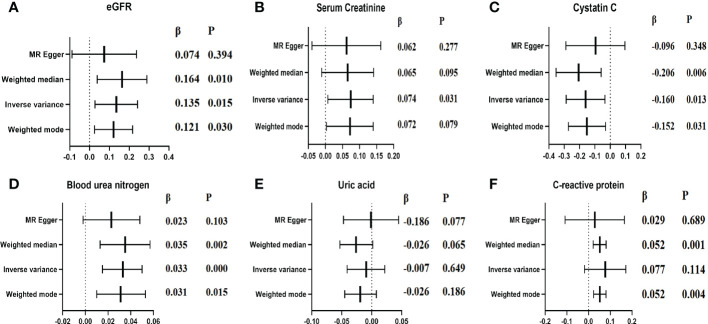
Causal estimates of genetically predicted lp(a) level on indexes of renal function. **(A)**: Causal estimates of genetically predicted lp(a) level in eGFR; **(B)**: Causal estimates of genetically predicted lp(a) level in Scr; **(C)**: Causal estimates of genetically predicted lp(a) level in Cystatin c; **(D)**: Causal estimates of genetically predicted lp(a) level in BUN; **(E)**: Causal estimates of genetically predicted lp(a) level in UA; **(F)**: Causal estimates of genetically predicted lp(a) level in CRP;.

**Figure 6:**
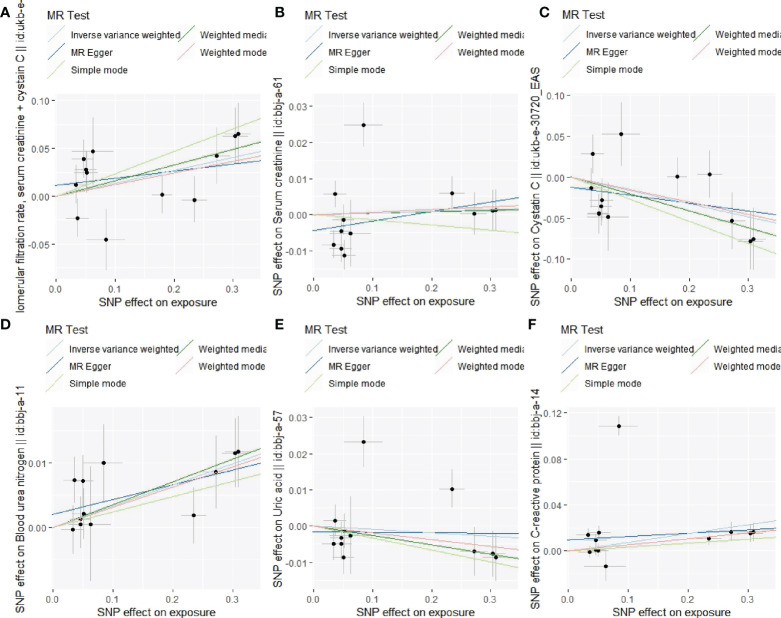
Associations of lp(a) variants with the indexes of renal function in different methods. **(A)**: Association of lp(a) variants with eGFR; **(B)**: Association of lp(a) variants with Scr; **(C)**: Association of lp(a) variants with cystatin C; **(D)**: Association of lp(a) variants with BUN; **(E)**: Association of lp(a) variants with UA; **(F)**: Association of lp(a) variants with C-reactive protein.

**Figure 5** elucidated the main MR results suggesting the causal association of lp(a) level with six indexes of renal function (eGFR, Scr, Cystatin C, BUN, UA, and C-reactive protein). The results suggested a causal relationship between increased lp(a) levels and four renal function indexes: a one unit higher lp(a) was associated with 13.5% higher eGFR (P=0.015), 7.4% higher Scr(P=0.031), 16.0% lower cystatin C (P=0.013), 3.3% higher BUN(P<0.001) in the inverse-variance weighted (IVW) analysis. A causal relationship was observed: one unit higher lp(a) was associated with 5.2% (P=0.004) higher C-reactive protein (β=0.052, P=0.004) in the weighted mode method. In contrast, no significant association was observed between increased lp(a) levels and UA (β= -0.007, P=0.649) in the inverse- IVW analysis. Three other MR methods had consistent observation (**Figures 5**, **6**).

The leave-one-out sensitivity analysis suggested that the association between lp(a) levels and 6 indexes of renal function were not considerably driven by any single SNP (**Supplementary Figure S6**). As seen in **Table 6**, pleiotropy bias did not suggest any chance of pleiotropy for all 6 outcomes (P>0.05); the heterogeneity tests were not significant for all 5 outcomes but not for the C-reactive protein (P Egger < 0.001, PIVW < 0.001).

**Table 6:** Horizontal pleiotropy.

Outcome	Horizontal pleiotropy			Heterogeneity statistics	
	beta	se	P	Q value-Egger	Q value-IVW
eGFR (serum creatinine + cystatin C)	0.012	0.012	0.352	0.114	0.108
Creatinine	0.003	0.008	0.765	0.915	0.951
Cystatin C	-0.012	0.014	0.404	0.124	0.128
Blood urea nitrogen	0.002	0.002	0.303	0.694	0.667
Uric acid	-0009	0.159	0.553	0.007	0.010
C-reactive protein	0.010	0.010	0.359	<0.001	<0.001

## Discussion

In this large-scale survey data of the CHCN-BTH study in 25,343 community-dwelling Chinese adults, the mean eGFR was 105.1 ml/min/1.73 m^2^ (IQR 95.1 to 115.7), and the mean lp(a) was 18.8 (SD 20.5). Lp(a) levels were significantly and independently associated with four renal function indexes (eGFR, Scr, BUN, CRPHS). Individuals with a higher lp(a) level had a lower eGFR level, and the association with eGFR was stronger in individuals with lp(a) under 14 mg/dL. Moreover, the association was similar in individuals with or without diabetes or hypertension. While the two-sample MR analysis showed a reverse causal association between genetically increased lp(a) level and increased eGFR level which contrasted with our observational results. MR data confirmed the causal association of other renal function indexes (Scr and BUN), which were consistent with our observational results. The results were vigorous in sensitivity tests with various instruments and statistical methods.

Dyslipidemia has been broadly reported to be associated with renal disease. Plasma lp(a) levels show a harmonization of lp(a) synthesis, which performs a function in the liver, and catabolism, which is considered to engage the kidney but is not fully understood (6). The acquired lp(a) anomaly in CKD patients seems to be the result of reduced lp(a) clearance (6). Higher lp(a) levels have been reported with a decline of eGFR, at both the earliest and the end stages of kidney failure (24–27). Lin J reported higher Lp(a) levels were associated with mild kidney impairment independently in nearly 2000 patients with TM2D without severe renal function (serum creatinine-based eGFR <60 ml/min/1.73 m^2^) (27). Mahboob Rahman demonstrated that baseline lp(a) level was not significantly associated with the consequent development of renal disease in nearly 4000 mixed-race CKD patients 48% of whom had diabetes mellitus (28). A Meta-Analysis that included eleven studies with 9304 type 2 diabetes patients showed that higher serum lp (a) is independently associated with a higher risk of diabetic nephropathy (7). A National Health and Nutrition Examination Survey indicated a plausible ethnic discrepancy in the association between eGFR and lp(a), lower eGFR category was poorly positively associated with serum lp(a) concentrations, notably in non-Hispanic blacks (25). This potentially suggests lp(a) metabolism in CKD progress, but more research is required to enhance the insight for understanding the relationship and its potential influence considering the wider complex interrelationship between Lp(a) and kidney disease, and more studies are required to demonstrate the role of lp(a) in kidney disease (6). Inconsistent research results on the role of the kidney in lp(a) metabolism occur with relatively small sample sizes in some research. Because of the extended range and skewed distribution of plasma lp(a) level, large-scale study participants must be explored to access solid evidence. In addition, most of the current research focuses on individuals with kidney disease or diabetes, and there are few studies based on the community-dwelling population.

We assumed that there might be a negative relationship between lp(a) and renal function in individuals without CKD, hypertension, or diabetes mellitus. Therefore, lp(a) could be a benefit in predicting the risk of CKD in individuals without hypertension or diabetes mellitus. Due to the lack of a large-scale community-based population study on the relationship between lp(a) level and renal function, particularly in the Chinese population, we conducted this cross-sectional and MR analysis on a total of 25,343 community-dwelling individuals in Beijing-Tianjin- Hebei region of northern China (9, 10). We not only explored the influence of lp(a) in the general population but also analyzed the effects of lp(a) in individuals with or without diabetes mellitus or hypertension. Our results suggested that elevated lp(a) was independently associated with the risk of slightly reduced renal, and other clinical indexes (eGFR, Scr, BUN, and CRPHS) in the general population, and the association with eGFR was non-linear which was stronger in individuals with relative low lp(a) level (under 14 mg/dL). Further stratified analysis showed that the associations between lp(a) and indexes of renal function were similar in individuals with or without diabetes mellitus or hypertension. No interactions have been observed between high lp(a) level (>30 mg/dL) and diabetes or hypertension on the risk of slightly reduced renal function. Another follow-up survey of community-based individuals in southern China including 6257 adults showed that the incidence of renal impairment increased with elevating lp(a) concentrations in the general population (8), which confirmed our findings. But Xuan’s study (8) indicated that the association of serum lp(a) and the incidence of reduced renal function was more prominent in patients with diabetes or hypertension, and a combined effect of diabetes and high lp(a) on the reduced renal function risk. But no significant association between lp(a) level and eGFR in individuals without hypertension was found in that study.

Although more evidence emerges, the causal effects of lipid components on CKD are still unclear, particular among the Chinese population. Thus, we used the two-sample MR analysis to assess the causal role of lp(a) levels determined by genetic variants and indexes of renal function in East Asian populations. We observed an association between genetically increased lp(a) and increased levels of serum creatinine and blood urea nitrogen, suggesting that increased lp(a) was causally associated with the damage of renal function, which was consistent with our observational results. Similar to our results, Jie Zheng (16) using MR approaches found reliable evidence for the causal effects of eight cardiometabolic-related risk factors including lp(a) on CKD in the European population. Intriguingly, genetically increased lp(a) was causally associated with the increased eGFR (estimated by creatinine and cystatin C) and decreased cystatin C, which indicated lp(a) benefited renal function. The MR result for eGFR as the outcome was contrary to our observational results and previous studies (6, 8). Because the serum creatinine and cystatin C were used to estimate the eGFR, the positive association between genetically determined lp(a) level and eGFR may be due to the reverse relationship for cystatin C as the outcome of MR analysis. We noted that Xuan’s study (8) only used serum creatinine to predict GFR, this may be the reason for the results of Xuan’s results were consistent with our observational study results and the MR analysis results with serum creatinine as the outcome, but not with the MR analysis with eGFR as the outcome. Serum cystatin C level is not only a sensitive marker for renal disease but also a predictive marker for CVD and inflammation. Ian H de Boer (29) reported that increased cystatin C concentrations were associated with greater TG and HDL-C concentrations in the multi-ethnic study of atherosclerosis. Seung-Hwan Lee (30) suggested that cystatin C level was significantly associated with biomarkers reflecting inflammation independent of renal function, and lipoprotein(a) showed significant correlations with cystatin C. However, the potential pathophysiological mechanism for the effect of lp(a) level on cystatin C warrants future research.

The biological mechanisms underlying the association between lp(a) and renal function damage remain uncertain. The fast decline in lp(a) level after transplantation is congruous with the metabolic role of the kidney in lp(a) catabolism (31). High lp(a) levels have also been found in several inflammatory conditions (32, 33). Different studies have indicated that lp(a) may raise inflammation in endothelial cells, monocytes, and macrophages, through the oxidized phospholipids (oxPL) that are combined with lp(a) (34, 35). Some studies suggested that a high level of lp(a) might participate in glomerular and tubulointerstitial damage (36, 37). Further experimental studies are required to explore the pathogenic mechanism of lp(a) abnormality with renal function, especially with cystatin C.

An advantage of this research was that the current research evaluated the associations of genetically calculated lp(a) levels with indexes of renal function in the Northern Chinese population with a relatively large sample size covering young to older adults. Second, we present evidence respecting a general community-based population instead of a specific disease population (i.e., CKD patients, hypertension patients, and diabetes patients) utilized in previous articles, which enhanced the generalizability of the conclusion. Third, given the results have an ethnic discrepancy, this study provides evidence to empower the precise conclusion about the Chinese population. Another advantage of the research is the use of the MR method based on several lp(a) level–related genetic variants as instruments and effects of genetic variants and outcomes from large open GWAS studies. MR method is an approach based on using genetic variants as instruments to calculate the causal effect of exposure to the disease (38). The potential bias is greatly diminished because genetic variation is not associated with other potential confounding factors which may influence observational studies (38). As far as we know, our study was the first to analyze the causal association between lp(a) and renal function in the East Asian population.

Some limitations of our study should be acknowledged. Self-reported demographic information, lifestyle characteristics, and disease status were obtained through a questionnaire, so there may be recall bias. Data for specific medication usage were not available in our study. While the absence of medication data may not affect the results, most lipid-lowering drugs have a limited effect on lp(a). We did not measure cystatin C, therefore, the associations of lp (a) with the cystatin C remain to be defined. The baseline survey data of the cohort were used in the present study, and thus the follow-up data result was absent. Unfortunately, the GWAS summary statistics data for CKD in East Asia for MR analysis were not available in our study.

In conclusion, serum lp (a) levels were significantly and independently associated with four renal function indexes (eGFR, Scr, BUN, CRPHS) in young, middle, and older Chinese adults regardless of diabetes or hypertension. Lp(a) showed causal effects on the causal association of three renal function indexes (Scr, cystatin C, and BUN) in East Asians. Individuals with a higher lp(a) level had a lower eGFR level, and the association with eGFR was stronger in individuals with lp(a) under 14 mg/dL. Taken together, these results highlight the attention of measurements of lp(a) for clinical practice of lp(a)-hyperlipoproteinemia and provide a vision for the future pattern of interventions to reduce the burden of renal function damage. Further studies are needed to explore the mechanism underlying lp(a) abnormality with renal function, especially with the renal function index cystatin C.

## Data availability statement

The genotyping data for this article are not publicly available to assure patient confidentiality and participant privacy. The data that support the findings of this study are available on request from the corresponding author (zlilyepi@ccmu.edu.cn).

## Ethics statement

The studies involving human participants were reviewed and approved by Ethics Committee of the Centre of Disease Control (IRB2017-003, CYCDPCIRB-20170830–1) and Capital Medical University (2018SY81). The patients/participants provided their written informed consent to participate in this study.

## Author contributions

Concept and design: LZ, YX. Collection, analysis, and interpretation of data: YX, HQ, BL, FW, FZ, CG. Stasiticical analysis: YX. Draft the manuscript: YX. Reviewed the manuscript LZ. All authors contributed to the article and approved the submitted version.

## Funding

This research was funded by the Natural Science Foundation of China (81973121 & 81373076), and the National Key Research and Development Program of China (2016YFC0900600/2016YFC0900603).

## Conflict of interest

The authors declare that the research was conducted in the absence of any commercial or financial relationships that could be construed as a potential conflict of interest.

## Publisher’s note

All claims expressed in this article are solely those of the authors and do not necessarily represent those of their affiliated organizations, or those of the publisher, the editors and the reviewers. Any product that may be evaluated in this article, or claim that may be made by its manufacturer, is not guaranteed or endorsed by the publisher.
