# Supplementary material for: Serum lipoprotein (a) associates with the risk of renal function damage in the CHCN-BTH Study: Cross-sectional and Mendelian randomization analyses
Source: Front Endocrinol (Lausanne). 2022 Nov 23;13:1023919. doi: 10.3389/fendo.2022.1023919 (PMC9727385; doi:10.3389/fendo.2022.1023919)
Supplement: Supplementary file 1 [file DataSheet_1.docx]

Supplementary Material

**
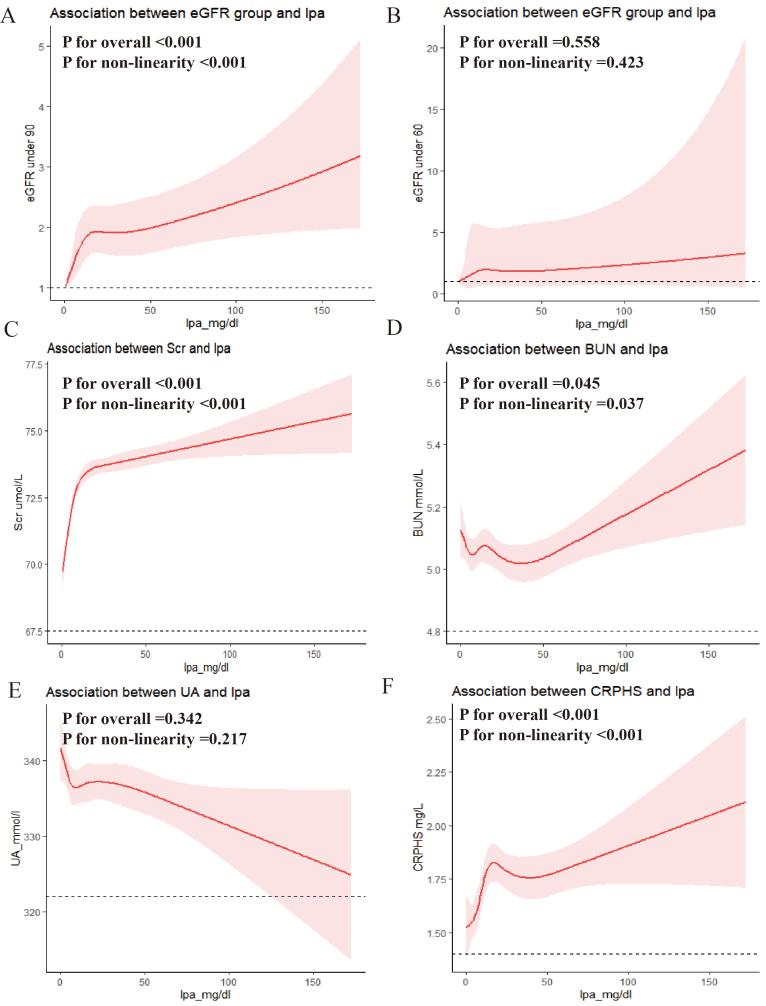
**

**Supplementary Figure S1**. The dose-response associations between lp(a) and index of renal function. A: lp(a) and eGFR as binary variable (eGFR under 90).; B: lp(a) and eGFR as binary variable (eGFR under 60); C: lp(a) and Scr; D: lp(a) and BUN; E: lp(a) and UA; F: lp(a) and CRPHS.


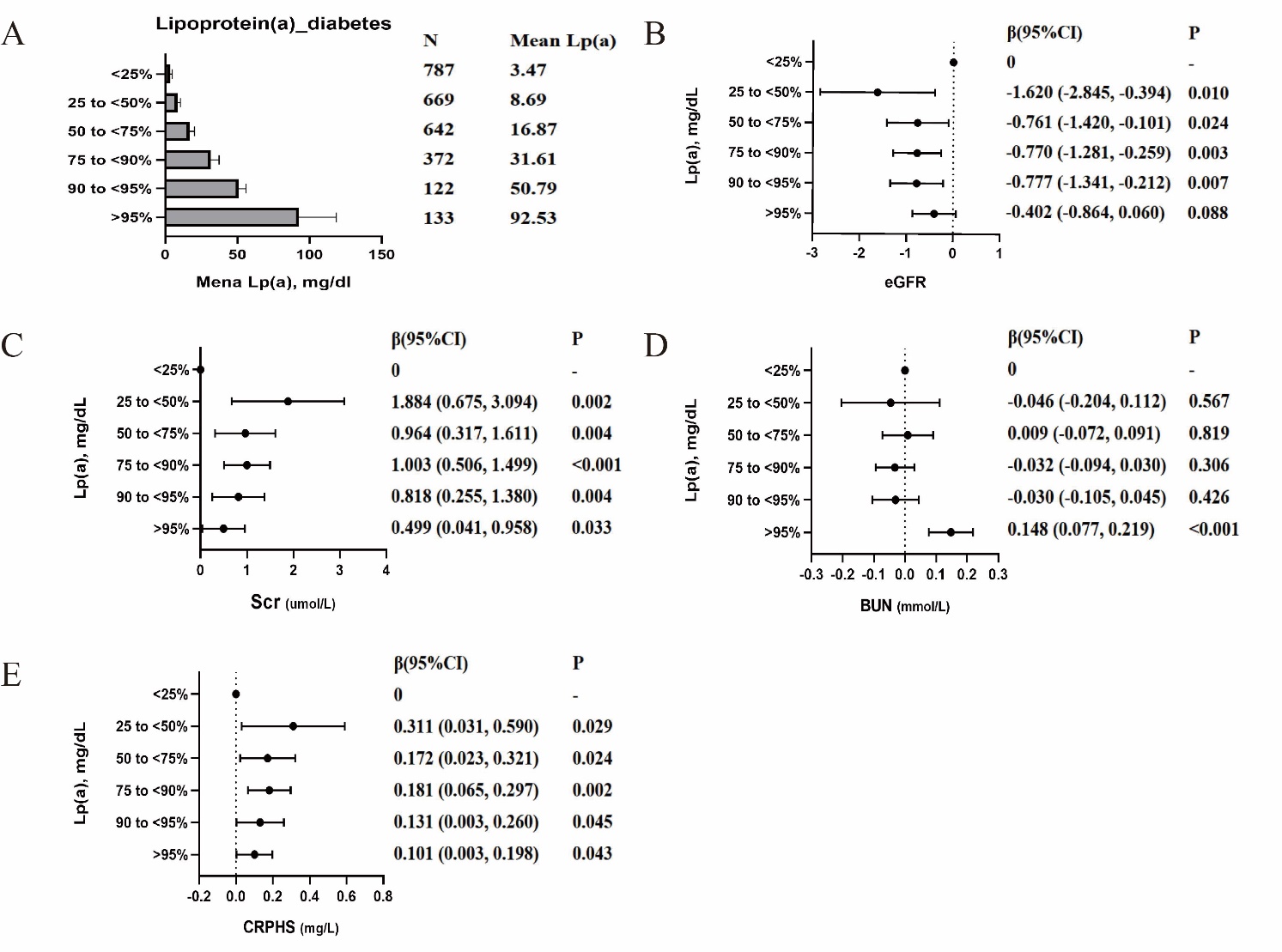


**Supplementary Figure S2**. The association between lp(a) (below 25^nd^ percentile, 25^nd^ to 50^th^, 50^th^ to 75^th^, 75^th^ to 95^th^, above 95^th^) with the index of renal function in people with diabetes. Each model was adjusted by potential confounders except for itself.


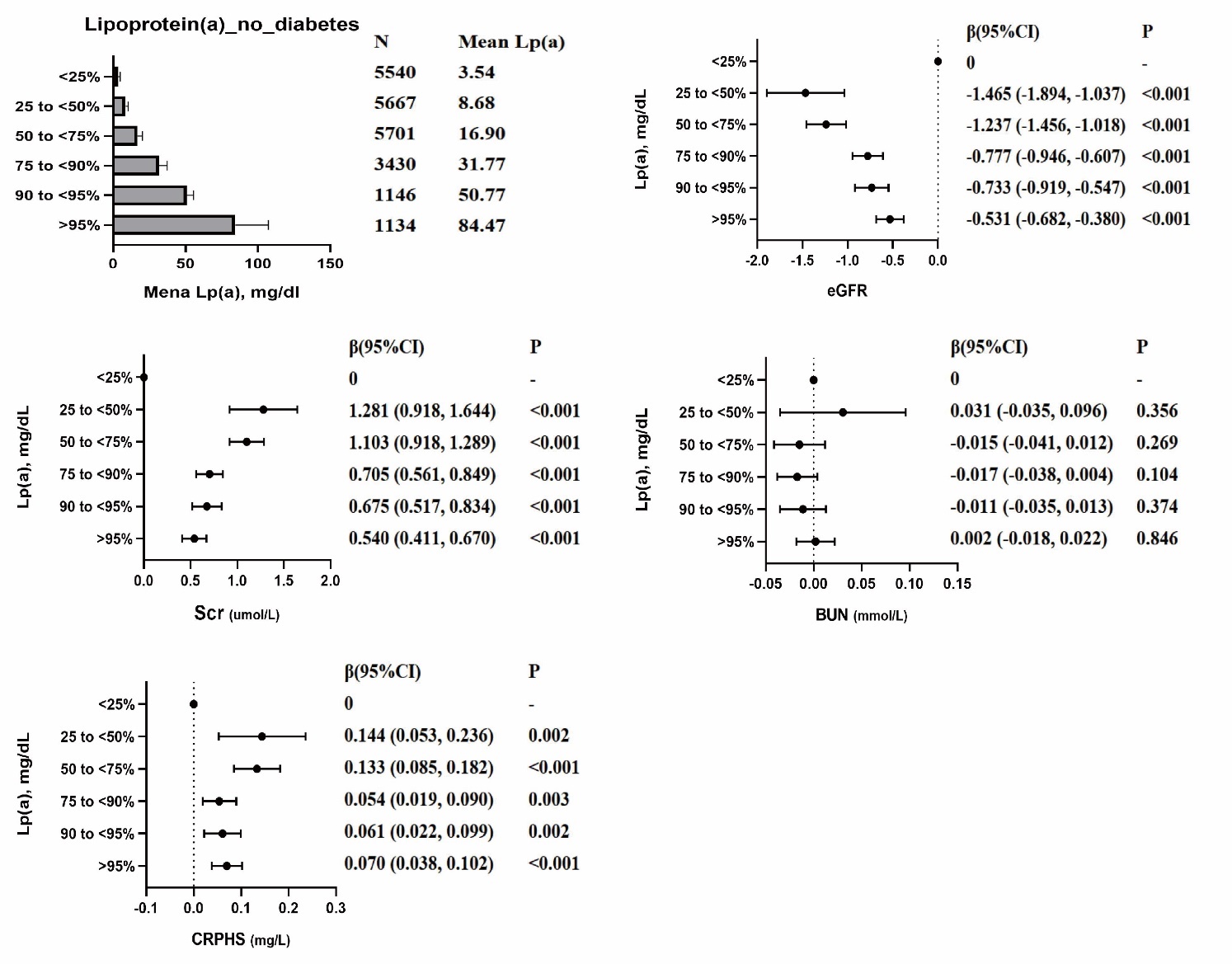


**Supplementary Figure S3**. The association between lpa (below 25^nd^ percentile, 25^nd^ to 50^th^, 50^th^ to 75^th^, 75^th^ to 95^th^, above 95^th^) with index of renal function in people without diabetes. Each model was adjusted by potential confounders except for itself.


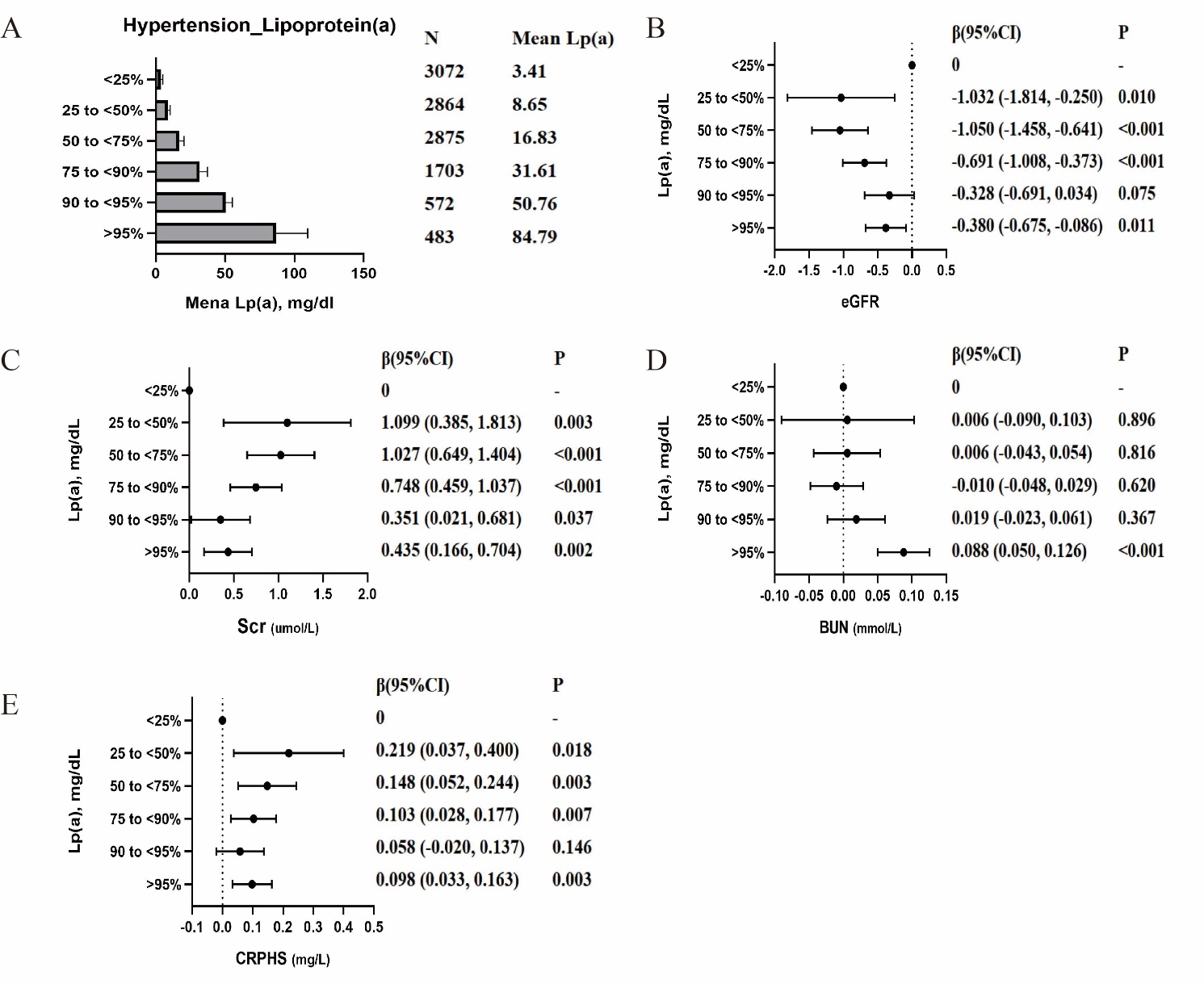


**Supplementary Figure S4**. The association between lpa (below 25^nd^ percentile, 25^nd^ to 50^th^, 50^th^ to 75^th^, 75^th^ to 95^th^, above 95^th^) with index of renal function in people with hypertension.Each model was adjusted by potential confounders except for itself.


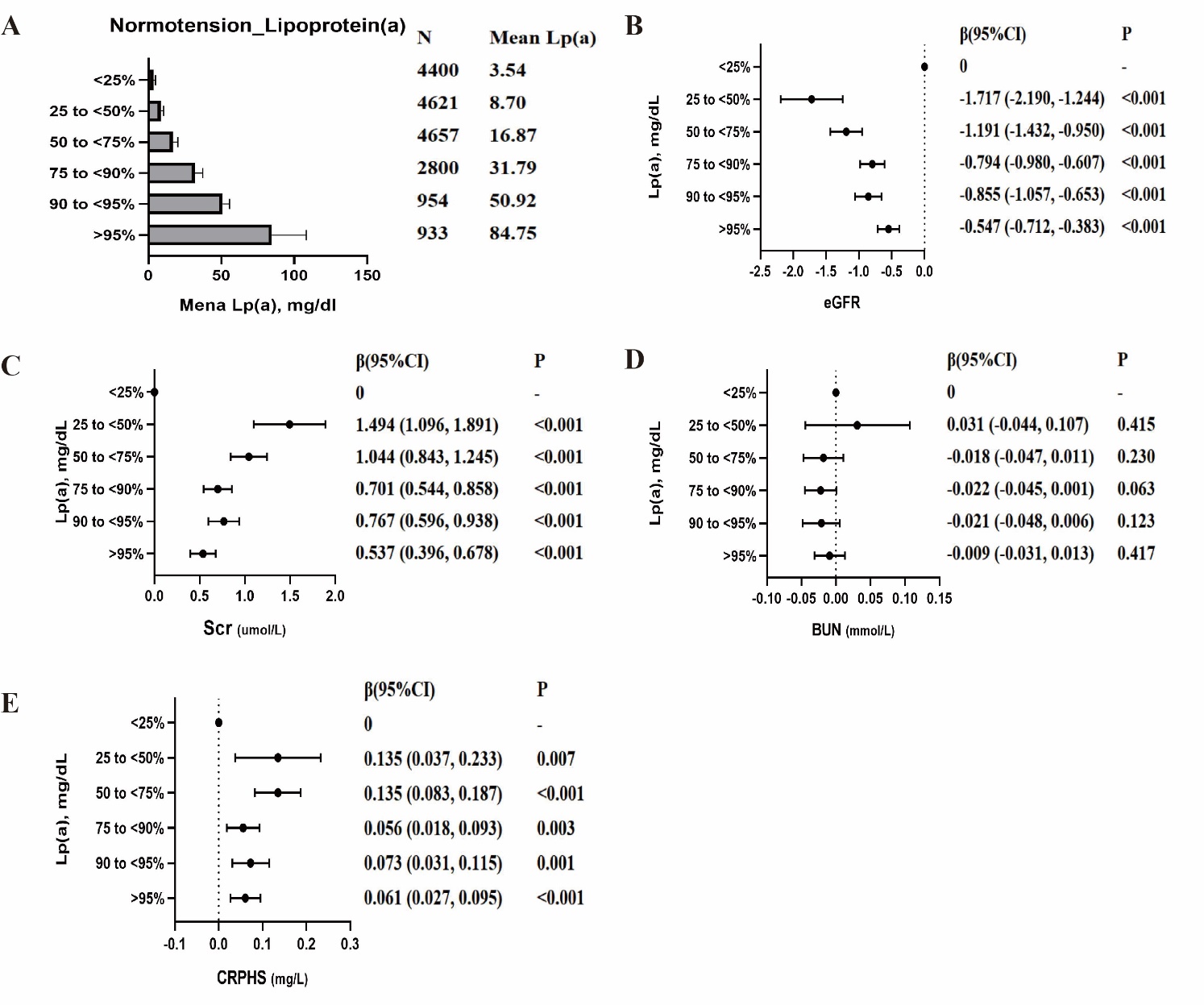


**Supplementary Figure S5**. The association between lp(a) (below 25^nd^ percentile, 25^nd^ to 50^th^, 50^th^ to 75^th^, 75^th^ to 95^th^, above 95^th^) with the index of renal function in people without hypertension. Each model was adjusted by potential confounders except for itself.


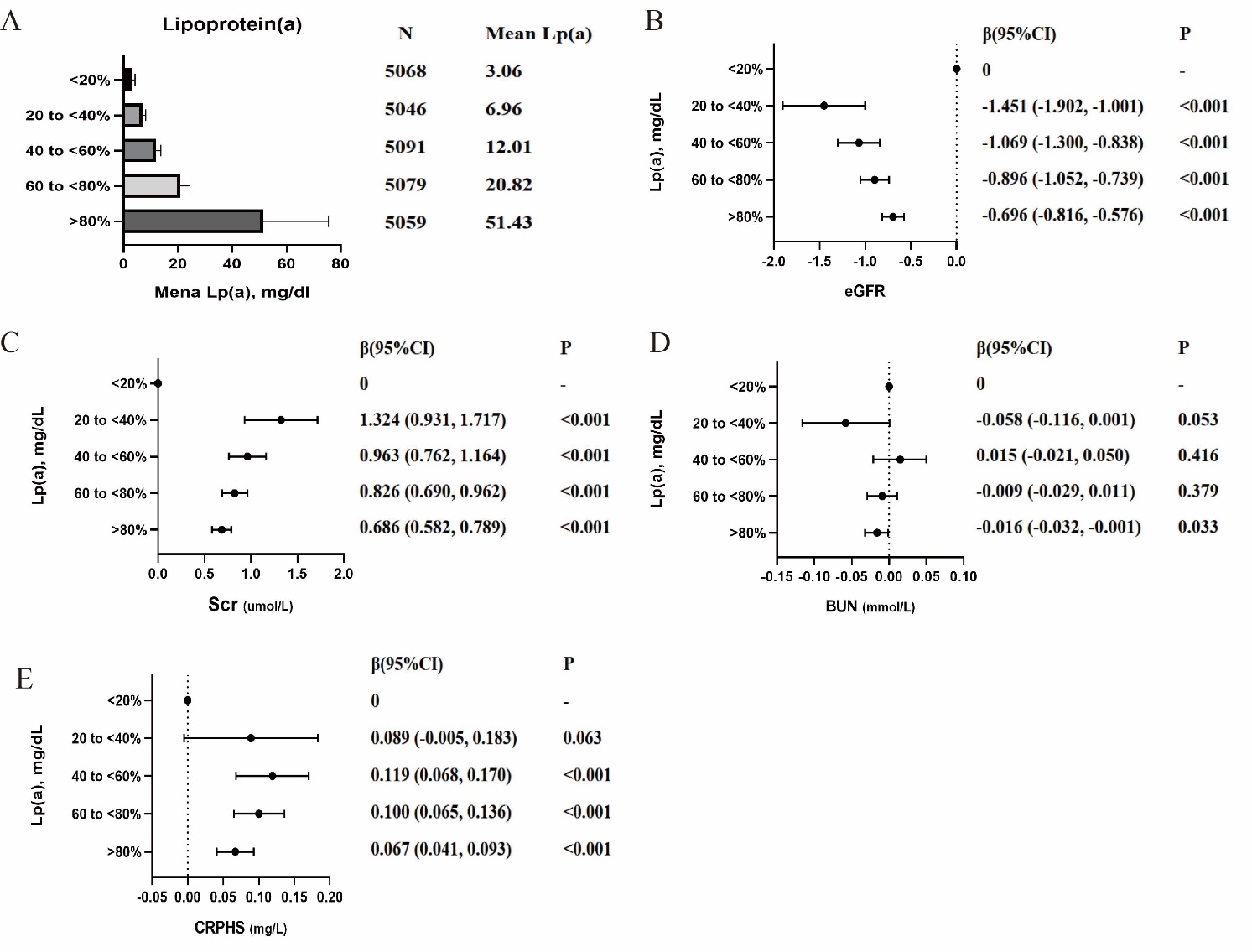


**Supplementary Figure S6**. The association between lp(a) (below 20^nd^ percentile, 20^nd^ to 40^th^, 40^th^ to 60^th^, 60^th^ to 80^th^, above 80^th^) with the index of renal function in total participants. Each model was adjusted by potential confounders except for itself.

**Supplementary Table S1**. Association of serum lp(a) concentrations with risk of renal function damage (below 20^nd^ percentile, 20^nd^ to 40^th^, 40^th^ to 60^th^, 60^th^ to 80^th^, above 80^th^).

|  |  | **Model 1** | | **Model 2** | |
| --- | --- | --- | --- | --- | --- |
| **eGFR<90** | **Cases, N (%)** | **OR (95%CI)** | **P** | **OR (95%CI)** | **P** |
| **Categorical** |  |  |  |  |  |
| < 20% | 597 (11.8) | Ref. | - | Ref. | - |
| 20 to < 40% | 752 (14.9) | 1.260 (1.108, 1.431) | <0.001 | 1.271 (1.117, 1.447) | <0.001 |
| 40 to < 60% | 819 (16.1) | 1/372 (1.209, 1.558) | <0.001 | 1.388 (1.220, 1.580) | <0.001 |
| 60 to < 80% | 926 (18.2) | 1.618 (1.428, 1.833) | <0.001 | 1.641 (1.445, 1.865) | <0.001 |
| >80% | 918 (18.1) | 1.537 (1.358, 1.743) | <0.001 | 1.603 (1.409, 1.823) | <0.001 |
| **P for trend** |  |  | <0.001 |  | <0.001 |

Mode1 was adjusted for age, gender, area, education, and BMI.

Model 2 was adjusted for age, gender, area, BMI, HDL-C, LDL-C, TG, TC, current smoking, alcohol, education, hypertension, diabetes.

**Supplementary Table S2.** The association between lp(a) (categorical variables) with the indexes of renal function after excluding individuals of eGFR under 90.

| Outcomes | lp(a) categories | β(95%CI) | P |
| --- | --- | --- | --- |
| Scr (umol/l) | <25% | Ref. | - |
|  | 25 to <50% | 1.170 (0.871, 1.469) |  |
|  | 50 to <75% | 0.797 (0.645, 0.950) | <0.001 |
|  | 75 to <90% | 0.500 (0.380, 0.619) | <0.001 |
|  | 90 to <95% | 0.531 (0.397, 0.664) | <0.001 |
|  | ≥95% | 0.399 (0.290, 0.509) | <0.001 |
|  |  |  |  |
| eGFR (ml/min/1.73 m^2^) | <25% | Ref. | - |
|  | 25 to <50% | -1.280 (-1.634, -0.926) | <0.001 |
|  | 50 to <75% | -0.887 (-1.070, -0.705) | <0.001 |
|  | 75 to <90% | -0.532 (-0.674, -0.390) | <0.001 |
|  | 90 to <95% | -0.554 (-0.711, -0.398) | <0.001 |
|  | ≥95% | -0.382 (-0.510, -0.254) | <0.001 |
|  |  |  |  |
| BUN (mmol/l) | <25% | Ref. | - |
|  | 25 to <50% | 0.030 (-0.031, 0.091) | 0.329 |
|  | 50 to <75% | -0.014 (-0.039, 0.010) | 0.239 |
|  | 75 to <90% | -0.018 (-0.036, 0.001) | 0.063 |
|  | 90 to <95% | -0.020 (-0.042, 0.001) | 0.061 |
|  | ≥95% | 0.008 (-0.011, 0.026) | 0.400 |
|  |  |  |  |
| CRPHS (mg/l) | <25% | Ref. | - |
|  | 25 to <50% | 0.189 (0.098， 0.280) | <0.001 |
|  | 50 to <75% | 0.145 (0.097, 0.192) | <0.001 |
|  | 75 to <90% | 0.069 (0.034, 0.103) | <0.001 |
|  | 90 to <95% | 0.060 (0.023, 0.098) | 0.002 |
|  | ≥95% | 0.070 (0.039, 0.101) | <0.001 |

Each model was adjusted by potential confounders except for itself.

**Supplementary Table S3.** The association between lp(a) (categorical variables) with the indexes of renal function after excluding individuals of eGFR under 60.

| Outcomes | Lp(a) categories | β (95%CI) | P |
| --- | --- | --- | --- |
| Scr (umol/l) | <25% | Ref. | - |
|  | 25 to <50% | 1.425 (1.081, 1.769) | <0.001 |
|  | 50 to <75% | 1.061 (0.886, 1.237) | <0.001 |
|  | 75 to <90% | 0.747 (0.611, 0.884) | <0.001 |
|  | 90 to <95% | 0.692 (0.541, 0.844) | <0.001 |
|  | ≥95% | 0.528 (0.405, 0.651) | <0.001 |
|  |  |  |  |
| eGFR (ml/min/1.73 m^2^) | <25% | Ref. | - |
|  | 25 to <50% | -1.560 (-1.961, -1.159) | <0.001 |
|  | 50 to <75% | -1.165 (-1.370, -0.960) | <0.001 |
|  | 75 to <90% | -0.790 (-0.949, -0.631) | <0.001 |
|  | 90 to <95% | -0.743 (-0.918, -0.568) | <0.001 |
|  | ≥95% | -0.509 (-0.650, -0.367) | <0.001 |
|  |  |  |  |
| BUN (mmol/l) | <25% | Ref. | - |
|  | 25 to <50% | 0.021 (-0.041, 0.082) | 0.510 |
|  | 50 to <75% | -0.014 (-0.039, 0.011) | 0.627 |
|  | 75 to <90% | -0.019 (-0.039, 0.001) | 0.064 |
|  | 90 to <95% | -0.014 (-0.036, 0.009) | 0.244 |
|  | ≥95% | 0.017 (-0.003, 0.036) | 0.091 |
|  |  |  |  |
| CRPHS (mg/l) | <25% | Ref. | - |
|  | 25 to <50% | 0.166 (0.078, 0.253) | <0.001 |
|  | 50 to <75% | 0.138 (0.093, 0.184) | <0.001 |
|  | 75 to <90% | 0.070 (0.036, 0.104) | <0.001 |
|  | 90 to <95% | 0.068 (0.031, 0.105) | 0.001 |
|  | ≥95% | 0.074 (0.044, 0.104) | <0.001 |

Each model was adjusted by potential confounders except for itself.

**Supplementary Table S4.** The association between lp(a) (categorical variables) with the indexes of renal function was stratified by gender.

|  |  | Male | | Female | |
| --- | --- | --- | --- | --- | --- |
|  |  | β (95%CI) | P | β (95%CI) | P |
| Scr (umol/l) | <25% | Ref. | - | Ref. | - |
|  | 25 to <50% | 1.547 (0.977, 2.118) | <0.001 | 1.044 (0.618, 1.470) | <0.001 |
|  | 50 to <75% | 1.321 (1.030, 1.612) | <0.001 | 0.796 (0.576, 1.017) | <0.001 |
|  | 75 to <90% | 0.815 (0.590, 1.041) | <0.001 | 0.626 (0.456, 0.796) | <0.001 |
|  | 90 to <95% | 0.870 (0.616, 1.123) | <0.001 | 0.524 (0.339, 0.708) | <0.001 |
|  | ≥95% | 0.656 (0.435, 0.877) | <0.001 | 0.458 (0.312, 0.603) | <0.001 |
|  |  |  |  |  |  |
| eGFR (ml/min/1.73 m^2^) | <25% | Ref. | - | Ref. | - |
|  | 25 to <50% | -2.087 (-2.832, -1.343) | <0.001 | -0.848 (-1.235, -0.461) | <0.001 |
|  | 50 to <75% | -1.639 (-2.018, -1.260) | <0.001 | -0.707 (-0.910, -0.505) | <0.001 |
|  | 75 to <90% | -0.972 (-1.267, -0.678) | <0.001 | -0.580 (-0.738, -0.423) | <0.001 |
|  | 90 to <95% | -1.029 (-1.356, -0.703) | <0.001 | -0.487 (-0.654, -0.320) | <0.001 |
|  | ≥95% | -0.705 (-0.989, -0.420) | <0.001 | -0.395 (-0.526, -0.263) | <0.001 |
|  |  |  |  |  |  |
| eGFR under 90 | <25% | 1.000 | - | 1.000 | - |
| (ml/min/1.73 m^2^) | 25 to <50% | 1.381 (1.180, 1.617) | <0.001 | 1.101 (0.927, 1.306) | 0.273 |
|  | 50 to <75% | 1.648 (1.409, 1.929) | <0.001 | 1.334 (1.125, 1.581) | 0.001 |
|  | 75 to <90% | 1.525 (1.272, 1.827) | <0.001 | 1.495 (1.237, 1.806) | <0.001 |
|  | 90 to <95% | 1.946 (1.506, 2.515) | <0.001 | 1.297 (0.977, 1.722) | 0.072 |
|  | ≥95% | 1.641 (1.241, 2.170) | 0.001 | 1.671 (1.292, 2.161) | <0.001 |
|  |  |  |  |  |  |
| BUN (mmol/l) | <25% | Ref. | - | Ref. | - |
|  | 25 to <50% | 0.049 (-0.044, 0.142) | 0.302 | -0.007 (-0.087, 0.073) | 0.864 |
|  | 50 to <75% | -0.011 (-0.047, 0.025) | 0.561 | -0.023 (-0.058, 0.012) | 0.205 |
|  | 75 to <90% | -0.020 (-0.048, 0.007) | 0.151 | -0.018 (-0.046, 0.010) | 0.198 |
|  | 90 to <95% | -0.001 (-0.034, 0.032) | 0.953 | -0.023 (-0.055, 0.008) | 0.145 |
|  | ≥95% | 0.043 (0.012, 0.073) | 0.006 | 0.005 (-0.020, 0.030) | 0.357 |
|  |  |  |  |  |  |
| CRPHS (mg/l) | <25% | Ref. | - | Ref. | - |
|  | 25 to <50% | 0.209 (0.078, 0.340) | 0.002 | 0.114 (-0.003, 0.230) | 0.056 |
|  | 50 to <75% | 0.182 (0.111, 0.252) | <0.001 | 0.098 (0.037, 0.159) | 0.002 |
|  | 75 to <90% | 0.081 (0.030, 0.132) | 0.002 | 0.058 (0.012, 0.104) | 0.013 |
|  | 90 to <95% | 0.116 (0.061, 0.172) | <0.001 | 0.028 (-0.022, 0.077) | 0.276 |
|  | ≥95% | 0.106 (0.058, 0.154) | <0.001 | 0.051 (0.012, 0.090) | 0.010 |

Each model was adjusted by potential confounders except for itself.

**Supplementary Table S5.** The association between lp(a) with the indexes of renal function was stratified by age.

|  |  | Young adults [age 18-35]  N=4860 | | Middle-aged adults [age 36-55]  N=10103 | | Older adults [age older than 55]  N=10380 | |
| --- | --- | --- | --- | --- | --- | --- | --- |
|  |  | β (95%CI) | P | β (95%CI) | P | β (95%CI) | P |
| Scr (umol/l) | <25% | Ref. | - | Ref. | - | Ref. | - |
|  | 25 to <50% | 1.797 (1.043, 2.550) | <0.001 | 1.046 (0.534, 1.559) | <0.001 | 0.983 (0.387, 1.578) | 0.001 |
|  | 50 to <75% | 1.275 (0.903, 1.648) | <0.001 | 0.839 (0.572, 1.106) | <0.001 | 0.896 (0.589, 1.204) | <0.001 |
|  | 75 to <90% | 0.816 (0.523, 1.109) | <0.001 | 0.654 (0.446, 0.863) | <0.001 | 0.543 (0.308, 0.778) | <0.001 |
|  | 90 to <95% | 0.912 (0.607, 1.217) | <0.001 | 0.582 (0.349, 0.815) | <0.001 | 0.571 (0.307, 0.835) | <0.001 |
|  | ≥95% | 0.429 (0.161, 0.696) | 0.002 | 0.589 (0.397, 0.781) | <0.001 | 0.406 (0.195, 0.616) | <0.001 |
|  |  |  |  |  |  |  |  |
| eGFR | <25% | Ref. | - | Ref. | - | Ref. | - |
| (ml/min/1.73 m^2^) | 25 to <50% | -2.432 (-3.521, -1.344) | <0.001 | -1.285 (-1.894, -0.676) | <0.001 | -0.760 (-1.369, -0.152) | 0.014 |
|  | 50 to <75% | -1.694 (-2.237, -1.151) | <0.001 | -0.986 (-1.303, -0.669) | <0.001 | -0.770 (-1.083, -0.456) | <0.001 |
|  | 75 to <90% | -1.068 (-1.490, -0.645) | <0.001 | -0.814 (-1.062, -0.566) | <0.001 | -0.395 (-0.636, -0.154) | 0.001 |
|  | 90 to <95% | -1.302 (-1.733, -0.872) | <0.001 | -0.597 (-0.875, -0.319) | <0.001 | -0.482 (-0.750, -0.214) | <0.001 |
|  | ≥95% | -0.591 (-0.969, -0.214) | 0.002 | -0.619 (-0.846, -0.392) | <0.001 | -0.294 (-0.508, -0.081) | 0.007 |
|  |  |  |  |  |  |  |  |
| eGFR under 90 | <25% | 1.000 |  | 1.000 |  | 1.000 |  |
|  | 25 to <50% | 2.426 (1.195, 4.925) | 0.014 | 1.187 (0.935, 1.506) | 0.159 | 1.203 (1.048, 1.380) | 0.009 |
|  | 50 to <75% | 2.473 (1.225, 4.898) | 0.011 | 1.579 (1.256, 1.985) | <0.001 | 1.369 (1.192, 1.572) | <0.001 |
|  | 75 to <90% | 3.210 (1.548, 6.656) | 0.002 | 1.782 (1.381, 2.299) | <0.001 | 1.311 (1.121, 1.534) | 0.001 |
|  | 90 to <95% | 3.208 (1.294, 7.949) | 0.012 | 1.796 (1.258, 2.562) | 0.001 | 1.395 (1.103, 1.763) | 0.005 |
|  | ≥95% | 1.090 (0.293, 4.056) | 0.897 | 1.868 (1.315, 2.654) | <0.001 | 1.493 (1.190, 1.874) | <0.001 |
|  |  |  |  |  |  |  |  |
| BUN (mmol/l) | <25% | Ref. | - | Ref. | - | Ref. | - |
|  | 25 to <50% | -0.077 (-0.212, 0.058) | 0.266 | 0.038 (-0.053, 0.129) | 0.414 | 0.069 (-0.033, 0.171) | 0.186 |
|  | 50 to <75% | -0.007 (-0.054, 0.040) | 0.777 | -0.019 (-0.053, 0.015) | 0.271 | 0.022 (-0.025, 0.069) | 0.355 |
|  | 75 to <90% | -0.025 (-0.061, 0.010) | 0.162 | -0.025 (-0.050, 0.001) | 0.057 | 0.008 (-0.029, 0.046) | 0.659 |
|  | 90 to <95% | -0.030 (-0.068, 0.009) | 0.128 | -0.018 (-0.047, 0.011) | 0.233 | 0.013 (-0.031, 0.058) | 0.552 |
|  | ≥95% | 0.001 (-0.032, 0.035) | 0.931 | 0.006 (-0.021, 0.032) | 0.671 | 0.050 (0.015, 0.086) | 0.006 |
|  |  |  |  |  |  |  |  |
| CRPHS (mg/l) | <25% | Ref. | - | Ref. | - | Ref. | - |
|  | 25 to <50% | 0.200 (0.007, 0.392) | 0.042 | 0.081 (-0.053, 0.215) | 0.235 | 0.196 (0.053, 0.340) | 0.007 |
|  | 50 to <75% | 0.187 (0.086, 0.288) | <0.001 | 0.065 (-0.002, 0.132) | <0.001 | 0.169 (0.089, 0.249) | <0.001 |
|  | 75 to <90% | 0.099 (0.024, 0.175) | 0.010 | 0.003 (-0.048, 0.054) | 0.921 | 0.092 (0.035, 0.150) | 0.002 |
|  | 90 to <95% | 0.064 (-0.012, 0.141) | 0.098 | 0.075 (0.016, 0.134) | 0.012 | 0.056 (-0.005, 0.117) | 0.074 |
|  | ≥95% | 0.044 (-0.023, 0.111) | 0.199 | 0.072 (0.023, 0.121) | 0.004 | 0.070 (0.023, 0.117) | 0.004 |

Each model was adjusted by potential confounders except for itself.
